# Supplementary material for: Exploration of Novel Biomarkers for Neurodegenerative Diseases Using Proteomic Analysis and Ligand-Binding Assays
Source: Biomedicines. 2024 Dec 9;12(12):2794. doi: 10.3390/biomedicines12122794 (PMC11673003; doi:10.3390/biomedicines12122794)
Supplement: Supplementary file 1 [file biomedicines-12-02794-s001.zip › biomedicines-3331442-supplementary.pdf]

Supplementary Table S1. Characteristics of the CSF samples from the validation cohort.

CSF	Control	AD	PD	MS	ALS
N	84	18	35	10	14
Age (mean±SD, years)	56.9±17.5	68.8±8.05	70.6±7.72	46.0±5.66	68.0±8.86
Sex (F/M/n.a.)	48/26/10	6/4/8	14/16/5	1/1/8	2/12/0

AD: Alzheimer's disease; ALS: amyotrophic lateral sclerosis; CSF: cerebrospinal fluid; F: female; M: male; MS: multiple sclerosis; n.a.: not applicable; PD: Parkinson's disease; SD: standard deviation.

Supplementary Table S2. Characteristics of the plasma samples from the validation cohort.

Plasma	Control	AD	PD	MS	ALS
N	11	14	13	9	5
Age (mean±SD, years)	n.a.	85.5±4.37	77.5±8.43	57	52.8±10.64
Sex (F/M/n.a.)	0/0/11	6/0/8	4/4/5	1/0/8	0/5/0

AD: Alzheimer's disease; ALS: amyotrophic lateral sclerosis; F: female; M: male; MS: multiple sclerosis; n.a.: not applicable; PD: Parkinson's disease; SD: standard deviation.

Supplementary Table S3. Most differentially expressed proteins (p < 1e-02) identified by an antibody microarray in CSF of patients with neurodegenerative diseases in comparison to controls.

Neurodegenerative disease	Protein	Antibody	logFC	Adj. P Val
Alzheimer's disease	LYVE1	ab2405	0.97	1.6e-06
	VCAM1	ab1792	0.79	4.6e-05
	CYTB	ab1241	0.75	4.6e-05
	TIMP1	ab1057	0.73	4.6e-05
	MERTK	ab2456	1.56	6.5e-05
	VEGF165b	ab2464	-1.76	1.2e-04
	MUC1	ab1087	-3.24	1.9e-04
	GRN	ab2704	1.32	2.1e-04
	MTOR	ab1153	-1.41	2.8e-04
	IL1AP	ab1770	0.88	4.8e-04
	MUC17	ab1267	-1.42	6.6e-04
	TNR1B	ab2455	0.67	7.7e-04
	TIMP1	ab1842	0.80	9.9e-04
	UROM	ab2694	-0.91	9.9e-04
	SLAF8	ab2129	-1.13	9.9e-04
	IBP4	ab0483	0.61	1.2e-03
	2A5D	ab1225	-1.65	1.2e-03
	RET4	ab1950	0.90	2.5e-03
	CFAD	ab1219	0.76	2.5e-03
	TNR14	ab1765	0.57	2.5e-03
	HMGB1	ab1215	-1.24	2.5e-03
	CO2	ab1216	0.78	2.6e-03
	MPIP3	ab1180	-1.65	2.6e-03
	HGFA	ab2253	0.72	2.7e-03
	IBP6	ab1979	0.58	2.9e-03
	ANFB	ab0774	-1.04	3.0e-03

	PLK1	ab1072	-1.25	3.0e-03
	CAMP	ab1184	1.15	3.4e-03
	COMP	ab2307	0.76	3.5e-03
	TGFR2	ab2475	0.74	3.5e-03
	IFG2	ab0482	0.59	3.5e-03
	CXCR5	ab1059	-1.13	3.5e-03
	GPI8	ab1317	-1.31	3.5e-03
	ACY1	ab2821	0.62	3.9e-03
	PARK7	ab2223	0.75	4.3e-03
	TNPO3	ab0583	-1.11	4.3e-03
	CATB	ab1172	-1.30	4.3e-03
	CD28	ab1559	-1.29	5.4e-03
	RARR2	ab2130	-0.93	5.8e-03
	CD14	ab1393	0.95	5.9e-03
	CTNB1	ab1183	-1.52	6.2e-03
	ING1	ab1032	-0.95	6.8e-03
	BEX3	ab0714	-0.93	7.0e-03
	AOXA	ab0441	-1.27	7.0e-03
	TMM54	ab0075	2.39	7.1e-03
	SODC	ab0644	-0.90	7.3e-03
	CAD13	ab1230	-1.09	7.3e-03
	IL26	ab1328	-1.28	7.3e-03
	G3P	ab1004	-1.37	7.3e-03
	RENI	ab2133	-1.67	7.3e-03
	A1BG	ab2811	0.72	7.4e-03
	CD38	ab1537	1.80	8.1e-03
	ANGI	ab1743	0.92	8.1e-03
	IGF1R	ab1995	-1.21	8.1e-03
	UB2R1	ab1305	-1.27	8.5e-03
	S10A8/9	ab1624	1.43	9.2e-03
	HAVR2	ab2067	-1.37	9.2e-03
	G3P	ab1332	-1.36	9.8e-03
**Parkinson's disease**	OSTP	ab1737	0.83	3.5e-03

Supplementary Table S4. Most differentially expressed proteins (p < 1e-02) identified by an antibody microarray in plasma of patients with neurodegenerative diseases in comparison to controls.

Neurodegenerative disease	Protein	Antibody	logFC	Adj. P Val
**Alzheimer's disease**	PGH2	ab0492	2.95	1.3e-03
	CORIN	ab1954	1.84	6.1e-03
**Parkinson's disease**	CD14	ab1393	1.43	3.4e-04
	TOP1	ab1298	1.69	8.6e-04
	IgE	ab1506	1.29	8.6e-04
	BASI	ab1487	1.30	2.4e-03
	Fc fusion with TNR1B	ab0520	1.63	2.6e-03
	ONCM	ab1885	0.97	3.6e-03
	CD8A	ab1376	1.31	4.7e-03

	CEAM6	ab1633	1.25	4.7e-03
	JAK2	ab0978	1.19	4.7e-03
	TNR1A	ab2332	1.11	4.7e-03
	IL13	ab1716	-1.25	4.7e-03
	CCCL7	ab1695	-1.00	5.2e-03
	MUC1	ab1087	1.73	5.6e-03
	IBP2	ab1835	1.09	5.6e-03
	S10A8/9	ab2724	0.88	5.6e-03
	TR13B	ab2250	1.20	6.1e-03
	IgE	ab1593	1.03	8.4e-03
	CD44	ab1437	1.00	8.3e-03
**Multiple sclerosis**	ANGT	ab2292	1.79	1.9e-04
	MMP9	ab1946	1.15	8.0e-04
	TR13B	ab2250	1.31	5.5e-03
	VEGF165b	ab2464	1.18	1.2e-03
